# Supplementary material for: Empirical methods for controlling false positives and estimating confidence in ChIP-Seq peaks
Source: BMC Bioinformatics. 2008 Dec 5;9:523. doi: 10.1186/1471-2105-9-523 (PMC2628906; doi:10.1186/1471-2105-9-523)
Supplement: Additional File 1 — A variety of html documents from the USeq web site detailing the available applications, their best usage, output file type descriptions, command line menus, etc. [file 1471-2105-9-523-S1.zip › USeqUserGuides/JavaDoc.zip!/JavaDoc/util/gen/MD5Crypt.html]

MD5Crypt


|  |  |  |
| --- | --- | --- |
| **Overview** \| **Package** \| **Class** \| **Use** \| **Tree** \| **Deprecated** \| **Index** \| **Help** | |  |
| **PREV CLASS**   **NEXT CLASS** | **FRAMES**    **NO FRAMES**     **All Classes** |  |
| SUMMARY: NESTED \| FIELD \| CONSTR \| METHOD | DETAIL: FIELD \| CONSTR \| METHOD |  |


---


## util.gen Class MD5Crypt

```
java.lang.Object
  util.gen.MD5Crypt
```

---

``` public final class MD5Crypt extends java.lang.Object ```

This class defines a method,
`crypt()`, which
takes a password and a salt string and generates an OpenBSD/FreeBSD/Linux-compatible
md5-encoded password entry.

Created: 3 November 1999

Release: $Name: $

Version: $Revision: 1.1 $

Last Mod Date: $Date: 2004/02/04 08:10:35 $

Java Code By: Jonathan Abbey, jonabbey@arlut.utexas.edu

Original C Version:

```
 ----------------------------------------------------------------------------
 "THE BEER-WARE LICENSE" (Revision 42):
  wrote this file.  As long as you retain this notice you
 can do whatever you want with this stuff. If we meet some day, and you think
 this stuff is worth it, you can buy me a beer in return.   Poul-Henning Kamp
 ----------------------------------------------------------------------------
```

Modified by Nix April 2005, just call MD5Crypt.crypt("password","salt"); returns crypted password, not magic or salt stuff.

---

**Constructor Summary**

`MD5Crypt()`


| **Method Summary** | |
| --- | --- |
| `static java.lang.String` | `crypt(java.lang.String password, java.lang.String salt)` |
| `static void` | `main(java.lang.String[] args)` |

**Methods inherited from class java.lang.Object**

`equals, getClass, hashCode, notify, notifyAll, toString, wait, wait, wait`

**Constructor Detail**

### MD5Crypt

```
public MD5Crypt()
```


**Method Detail**

### main

```
public static void main(java.lang.String[] args)
```

---


### crypt

```
public static final java.lang.String crypt(java.lang.String password,
                                           java.lang.String salt)
```


---


|  |  |  |
| --- | --- | --- |
| **Overview** \| **Package** \| **Class** \| **Use** \| **Tree** \| **Deprecated** \| **Index** \| **Help** | |  |
| **PREV CLASS**   **NEXT CLASS** | **FRAMES**    **NO FRAMES**     **All Classes** |  |
| SUMMARY: NESTED \| FIELD \| CONSTR \| METHOD | DETAIL: FIELD \| CONSTR \| METHOD |  |


---
